# Applying internal coordinate mechanics to model the interactions between 8R-lipoxygenase and its substrate

**DOI:** 10.1186/1471-2105-11-S6-S2

**Published:** 2010-10-07

**Authors:** Shuju Bai, Tianchuan Du, Ebrahim Khosravi

**Affiliations:** 1 Department of Computer Science, Southern University and A&M College, Baton Rouge, LA 70813, USA

## Abstract

**Background:**

Lipoxygenases (LOX) play pivotal roles in the biosynthesis of leukotrienes and other biologically active potent signalling compounds. Developing inhibitors for LOX is of high interest to researchers. Modelling the interactions between LOX and its substrate arachidonic acid is critical for developing LOX specific inhibitors. Currently, there are no LOX-substrate structures. Recently, the structure of a coral LOX, 8R-LOX, which is 41% sequence identical to the human 5-LOX was solved to 1.85Å resolution. This structure provides a foundation for modelling enzyme-substrate interactions.

**Methods:**

In this research, we applied a computational method, Internal Coordinate Mechanics (ICM), to model the interactions between 8R-LOX and its substrate arachidonic acid. Docking arachidonic acid to 8R-LOX was performed.  The most favoured docked ligand conformations were retained. We compared the results of our simulation with a proposed model and concluded that the binding pocket identified in this study agrees with the proposed model partially.

**Results:**

The results showed that the conformation of arachidonic acid docked into the ICM-identified docking site has less energy  than that docked into the manually defined docking site for pseudo wild type 8R-LOX.  The mutation at I805 resulted in no docking pocket found near Fe atom. The energy of the arachidonic acid conformation docked into the manually defined docking site is higher in mutant 8R-LOX than in wild type 8R-LOX. The arachidonic acid conformations are not productive conformations.

**Conclusions:**

We concluded that, for the wild type 8R-LOX, the conformation of arachidonic acid docked into the ICM-identified docking site is more stable than that docked into the manually defined docking site. Mutation affects the structure of the putative active site pocket of 8R-LOX, and leads no docking pockets around the catalytic Fe atom.  The docking simulation in a mutant 8R-LOX demonstrated that the structural change due to the mutation impacts the enzyme activity. Further research and analysis is required to obtain the 8R-LOX-substrate model.

## Background

Lipoxygenases (LOX) are non-heme iron dioxygenases that catalyze the stereo- and regio- specific formation of fatty acid hydroperoxides from polyunsaturated fatty acids, which are commonly found in plants and animals [1,2].  Human lipoxygenases play pivotal roles in the biosynthesis of leukotrienes and other biologically active potent signalling compounds in the inflammatory response [3]. Due to this, human lipoxygenases are targets for developing inhibitors to modulate the effects of the potent signalling compounds [4,5].  Leukotrienes are derived from arachidonic acid, which is the substrate of lipoxygenase [6].   An understanding of substrate arachidonic acid recognition is helpful in the design of enzyme-specific inhibitors [7]. However, there are no animal LOX structures available to provide a model for how the substrate binds in the active site.  The structure of a coral lipoxygenase, 8R-LOX, from *Plexaura homomalla,* with 41% identical to a human lipoxygenase, 5-LOX, was recently solved to 1.85 Å [7]. There is no human isozyme with better than 40% sequence identity to human 5-LOX. The high resolution structure of 8R-LOX can provide a strong foundation for modelling enzyme-substrate interactions.  

Many computational methods have been used to model the interactions between enzyme and substrate. Internal Coordinate Mechanics (ICM) is one of the computational methods used by researchers to predict enzyme-substrate interactions. ICM is a stochastic global optimization methodology with biased probability Monte Carlo procedure which can be used to effectively model substrate docking and predict structure [8-17]. It has been used to identify the active site of enzyme and acknowledged to be an accurate predictive tool of binding geometry today [13,18]. 

In this research, we will use the high resolution structure of 8R-LOX as the model protein and apply ICM to model how the substrate arachidonic acid, interacts with 8R-LOX. This research will lead us to develop substrate-LOX models for the lipoxygenase superfamily and facilitate the development of specific anti-LOX inhibitors.

## Methods

### Retrieval of high resolution structure of 8R-LOX and pre-processing

The high resolution 1.85Å structure of pseudo-wild type 8R-LOX (psWT)  and 1.9Å structure of a deletion mutant of psWT 8R-LOX (I805W:psWT) were obtained from RCSB Protein Data Bank with access code 3FG1 and 3FG3, respectively [19]. ICM Pro version 3.6 was used for docking arachidonic acid into the protein. 

**Figure 1:**
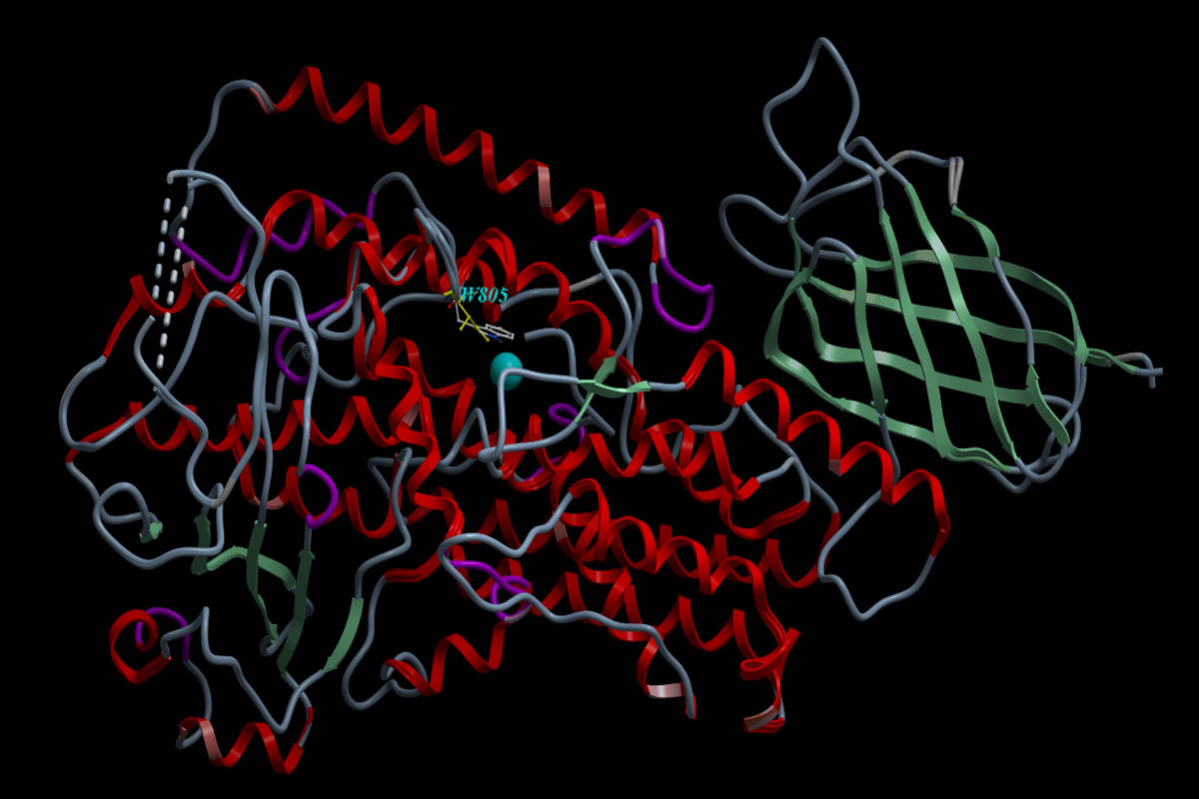
**The 1.85Å resolution crystal structure of pseudo-wild type 8R-LOX and the 1.9Å resolution crystal structure of the mutant I805W:psWT 8R-LOX used in docking simulation.** The structure of I805W:psWT superimposes on the structure of psWT 8R-LOX. Their structure is nearly identical except that in the area of the mutation.  W805 is marked out in the figure. The catalytic Fe atom is in blue.

First, the pdb files of psWT and I805W:psWT were converted into ICM objects, by optimizing hydrogens, optimizing side-chains and deleting water molecules. Since there are four identical molecules in psWT and I805W:psWT and we would need only one to perform the docking, we removed three of them to avoid the noises and inconveniences caused by the existence of the other three molecules. For the same purpose, the unrelated small molecules were also removed from the ICM objects of psWT and I805W:psWT (Figure 1).

### Docking arachidonic acid into 8R-LOX

ICM Pro version 3.6 was used to perform docking the substrate, arachidonic acid into psWT and I805W:psWT. We first set psWT and I805W:psWT as the receptors and found the potential ligand binding pockets in psWT and I805W:psWT.  Tolerance value we used was 3 to avoid missing any docking pockets (the default value is 5).

Of the potential ligand binding pockets found, the one which is closest to the catalytic Fe was selected to perform the docking. We also manually defined a docking site for psWT and I805W:psWT based on the binding site proposed by Neau [7]. This manually defined docking site includes 13 residues (Table 1). 

**Table 1:** Residues of the ICM-identified docking site for psWT and residues of the manually defined docking site for psWT and I805W:psWT 8R-LOX

No.	psWT (Manually defined)	I805W:psWT (Manually defined)	psWT (ICM-identified)
1	R555	R555	Q753
2	L758	L758	L754
3	L763	L763	H757
4	E766	E766	L758
5	I796	I796	H762
6	I799	I799	I796
7	G800	G800	D797
8	R801	R801	G800
9	L804	L804	L804
10	***I805***	***W805***	I810
11	I999	I999	V811
12	L1000	L1000	L815
13	I1066	I1066	T996
14			L1000
15			I1066

There are 13 residues for both manually defined docking sites in psWT and I805W:psWT 8R-LOX. The mutation residue is bolded and italicized. In psWT 8R-LOX, the 10^th^ residue is I805 while the mutant has W805. There are 15 residues for the ICM-identified docking site in psWT. The 6 common residues for the ICM-identified docking site and the manually defined docking site are underlined.

Reviewing and adjusting docking site was done. Then the receptor map was generated. This step was to construct energy map of the environment within the docking area box. Interactive docking was performed for the chosen docking pocket and manually defined docking pocket. The thoroughness which represents the length of simulation was set as 1 as suggested by ICM Pro.  Properties of conformations were saved for viewing and analysis.  

## Results

### Docking of arachidonic acid into psWT 8R-LOX

**Figure 2:**
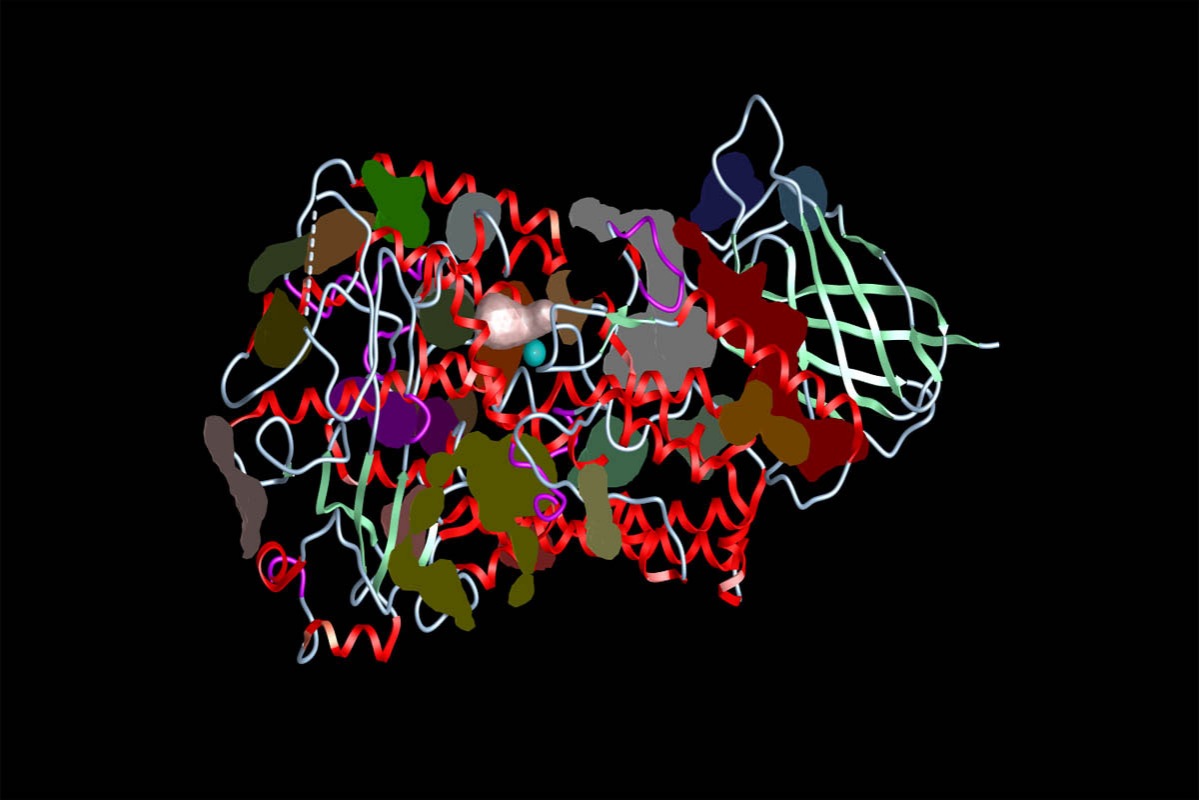
**Distribution of the 26 potential ligand binding pockets of psWT 8R-LOX.** The pockets are marked in different colours. The pocket for simulating the substrate docking is marked in pink. The catalytic Fe atom is in blue.

With tolerance value 3, we found 26 potential ligand binding pockets (Figure 2). Of the 26 potential ligand binding pockets, the one which was closest to Fe atom and located in the target area was chosen to dock arachidonic acid into psWT 8R-LOX (Figure 2).  All other binding pockets were not considered for doing docking because either they were too far from the catalytic Fe atom or the volume was too big to properly accommodate the small ligand, arachidonic acid.  The volume of the selected binding pocket is 171.162 Å^3^, and the radius of it is 3.4Å.  This pocket is composed of 15 residues plus Fe atom. The 15 residues are Q753, L754, H757, L758, H762, I796, D797, G800, L804, I810, V811, L815, T996, L1000 and I1066 (Table 1). 

**Figure 3:**
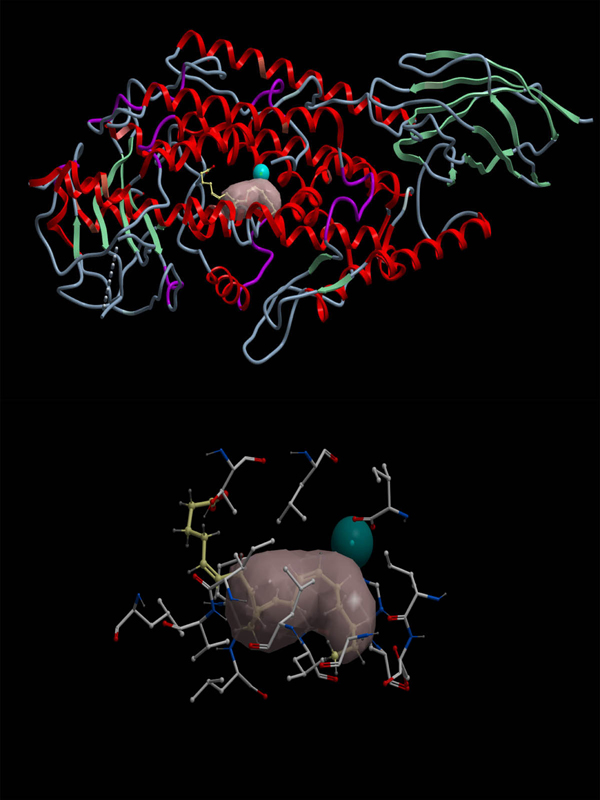
**The conformation of arachidonic acid docked into the ICM-identified docking site of psWT 8R-LOX with lowest energy.** Upper: An overall view of the conformation of arachidonic acid. Lower: A close up view of the conformation of acrachidonic acid.  The arachidonic acid is in yellow, the carboxyl terminal of it is in red. The catalytic Fe atom is in blue. The energy for this conformation is -63.623kcal/mol.

The docking simulation resulted in seventy-three conformations of arachidonic acid docked in the psWT 8R-LOX. The conformation of arachidonic acid with the lowest energy (-63.623kcal/mol) is shown in Figure 3. From the conformation, we can see arachidonic acid is docked into the binding pocket with carboxyl terminal pointing inside of psWT 8R-LOX.  However, there are other conformations with the carboxyl terminal pointing to the outside of psWT 8R-LOX although the energy is high. 

**Figure 4:**
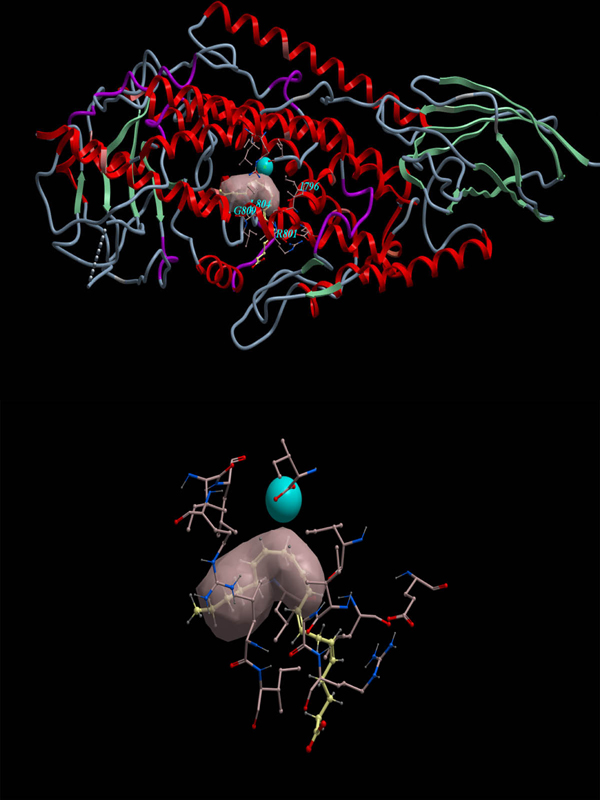
**The conformation of arachidonic acid docked into the manually defined docking site of psWT 8R-LOX.** Upper: An overall view of the conformation of arachidonic acid. Lower: A close up view of the conformation of acrachidonic acid.  The arachidonic acid is in yellow, the carboxyl terminal of it is in red. The catalytic Fe atom is in blue. The energy for this conformation is 2.706kcal/mol.

Interactive docking of arachidonic acid into the manually defined docking site (Table 1) resulted in seventy-six conformations of arachidonic acid docked into the psWT 8R-LOX. We found that the conformation of arachidonic acid with the lowest energy is not located around the Fe atom. Putting together information about location, energy, and feasibility, we identified one conformation as the most favoured conformation (Figure 4). This conformation is located near the Fe and with low energy (2.706kcal/mol). In this conformation, the carboxyl terminal of arachidonic acid points to outside of 8R-LOX, and the methyl C terminal is located inside of the psWT 8R-LOX.

### Docking of arachidonic acid to mutant I805W:psWT 8R-LOX

**Figure 5:**
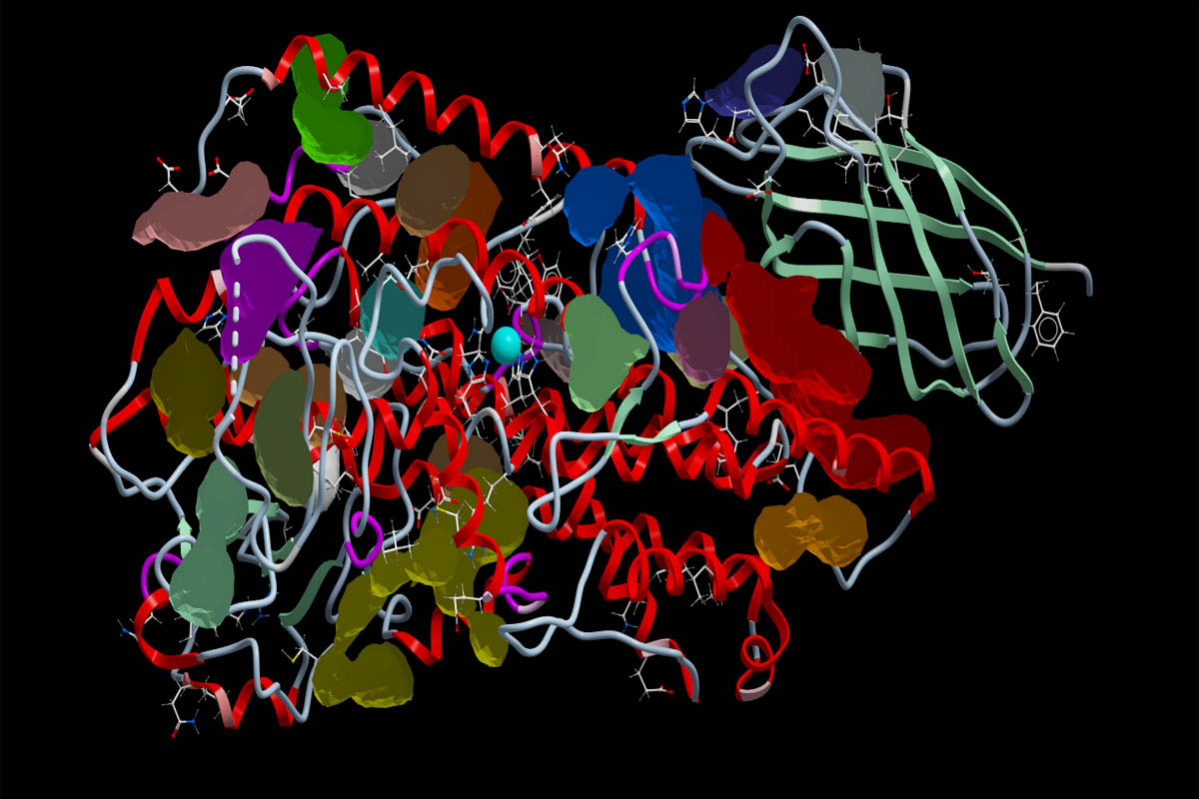
**The 25 potential ligand binding pockets of I805WT:psWT 8R-LOX.** Pockets are patches in various colours.  No pocket is located around the catalytic Fe atom. The catalytic Fe atom is in blue.

We found twenty-five potential ligand binding pockets in I805W:psWT 8R-LOX (Figure 5). There are none of these pockets enclosing the catalytic Fe atom. Data shows that the closest pocket to Fe atom is as far as 4.5Å from the catalytic Fe atom, not containing Fe atom. Since no pocket sits around Fe atom, no ICM docking was performed for any of these 25 pockets.  

**Figure 6:**
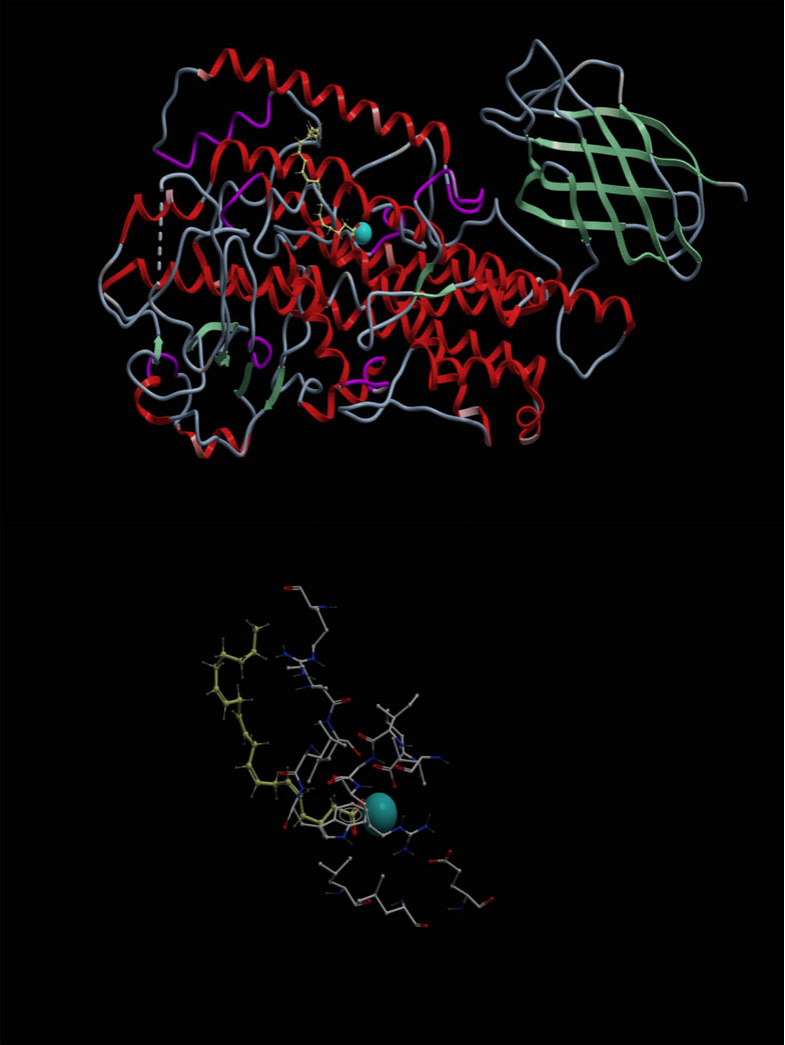
**The conformation of arachidonic acid docked into the manually defined docking site of I805WT:psWT 8R-LOX.** Upper: An overall view of the conformation of arachidonic acid. Lower: A close up view of the conformation of acrachidonic acid.The arachidonic acid is in yellow, the carboxyl terminal of it is in red. The catalytic Fe atom is in blue. The energy for this conformation is 39.947kcal/mol.

However, we did run ICM docking using the manually defined docking site for I805W:psWT (Table 1). Interactive docking simulation for the manually defined docking site resulted in 73 conformations of arachidonic acid docked to I805W:psWT 8R-LOX. The most favoured arachidonic acid conformation is near the Fe atom, 3.1 Å, and has the lowest energy, 39.947kcal/mol (Figure 6).

## Discussion

### Conformations of arachidonic acid docked into psWT 8R-LOX

Comparison of the conformations of arachidonic acid docked into the ICM-identified docking site and that docked into the manually defined docking site shows that the location of arachidonic acid for both conformations is overlapped.  However, there is difference between the two conformations. In the conformation of arachidonic acid docked into the ICM-identified docking site, the carboxyl terminal of arachidonic acid points inside of 8R-LOX, while in the conformation of arachidonic acid docked into the manually defined docking site,  the carboxyl terminal of arachidonic acid points outside of 8R-LOX (Figures 3 and 4). 

The conformation of arachidonic acid in Figure 4 implies a possible access for arachidonic acid to Fe atom from ambience. However, if we compare the energy for the two conformations, the conformation of arachidonic acid in Figure 3 is much lower than that in Figure 4, indicating the conformation of arachidonic acid in Figure 3 is more stable and favoured.  We realized that there are 6 residues in common for ICM-identified docking pocket and the manually defined docking pocket. They are L758, I796, G800, L804, L1000, and I1066.  These common residues explain why the locations of the two conformations are overlapped.  The common residues for the two docking pockets indicate that the binding site proposed by Neau et al [7] makes sense. Our simulation confirmed the proposed binding site partially.  Further simulation has been designed to verify/modify the proposed binding site in 8R-LOX.  

### Conformations of arachidonic acid docked into psWT 8R-LOX and I805W:psWT 8R-LOX

We found that 25 potential docking pockets were identified in I805W:psWT (Figure 5), which is 1 less than that in psWT 8R-LOX (Figure 2).  We can find that the pockets in Figure 2 and Figure 5 are basically the same except the area around Fe atom, indicating that the mutation at I805 changed the 3-D structure of the area around the catalytic Fe atom in 8R-LOX. The interesting thing is that there is none of the 25 pockets containing the catalytic Fe atom. This tells us that the mutation made at I805 caused a fundamental change in the binding site of 8R-LOX, leaving no docking pocket around Fe atom.  

The docking results for the manually defined docking site show that the energy of the arachidonic acid conformation in I805W:psWT, 39.947kcal/mol (Figure 6) is much greater than that in the  psWT 8R-LOX, 2.706kcal/mol (Figure 4). This tells us that the arachidonic acid conformation docked into the mutant 8R-LOX is much less stable than that docked into the wild-type 8R-LOX. The structure of the manually defined binding pocket is changed by the mutation, causing the binding ability of 8R-LOX for arachidonic acid weakened, the binding of arachidonic acid to 8R-LOX not stable, and therefore the activity of the enzyme reduced. This consists with the experimental data that mutations significantly impact enzyme activity [7].

### Productivity of arachidonic acid conformations

We analyzed the arachidonic acid conformations for all the docking simulations.  We found that no docking simulation produces productive conformation considering the stereo- and region- specificity of the product and the process of catalysis. The 10th carbon atom in all arachidonic acid conformations does not position against the catalytic Fe atom (Figures 3, 4, 6).  In the catalysis,  the first step of the reaction is hydrogen abstraction at the 10th carbon of a pentadiene moiety of arachidonic acid by the activated Fe^3+^[14] to produce a free radical intermediate, which requires the 10th carbon to face to Fe atom. All our arachidonic acid conformations do not show this.  The reason could be that we removed all the water molecules before doing the docking. However, in the hydrogen abstraction at the 10^th^ carbon of arachidonic acid, a water molecule is required [1,6]. We will perform the docking simulation with the water molecule present and hope to obtain productive conformations. 

## Conclusions

We applied the computational method “Internal Coordinate Mechanics” in docking arachidonic acid to 8R-LOX.  The results showed that, for the wild type 8R-LOX, the conformation of arachidonic acid docked into ICM-identified docking site is more stable than that docked into the manually defined docking site. Mutation at I805 affects the structure of the putative binding site of 8R-LOX, which leads no docking pockets around Fe atom.  The docking simulation in I805W:psWT 8R-LOX explains and consists with the experimental data presented by Neau [7]. Further research and analysis is required to obtain productive conformations, identify the binding site of 8R-LOX and model the interactions between arachidonic acid and 8R-LOX.  

## List of abbreviations used

LOX: Lipoxygenase; ICM: Internal coordinate mechanics; psWT- Pseudo-wild type 8R-LOX, I805W; psWT: A deletion mutant of psWT 8R-LOX.

## Competing interests

The authors declare that they have no competing interests.

## Authors' contributions

SB conceived of the study, and participated in its design and coordination and drafted the manuscript. TD performed the docking simulation, helped to draft the manuscript. EK participated in the design.  All authors read and approved the final manuscript.
